# Variation in the Current Use of Technology to Support Diabetes Management in UK Hospitals: Results of a Survey of Health Care Professionals

**DOI:** 10.1177/19322968231161076

**Published:** 2023-03-22

**Authors:** Alistair Lumb, Shivani Misra, Gerry Rayman, Parizad Avari, Daniel Flanagan, Pratik Choudhary, Ketan Dhatariya

**Affiliations:** 1 Oxford Centre for Diabetes, Endocrinology and Metabolism, Churchill Hospital, Oxford, UK; 2 Department of Metabolism, Digestion and Reproduction, Imperial College London, London, UK; 3 Department of Diabetes and Endocrinology, Imperial College Healthcare NHS Trust, London, UK; 4 Ipswich Diabetes Centre, East Suffolk and North Essex NHS Foundation Trust, Ipswich, UK; 5 Department of Endocrinology, University Hospital Plymouth, Plymouth, UK; 6 Diabetes Research Centre, University of Leicester, Leicester, UK; 7 Elsie Bertram Diabetes Centre, Norfolk and Norwich University Hospitals NHS Foundation Trust, Norwich, UK; 8 Norwich Medical School, University of East Anglia, Norwich, UK

**Keywords:** diabetes mellitus, hospital care, technology, continuous glucose monitoring, insulin pump

## Abstract

**Background:**

There has been a significant increase in the use of wearable diabetes technologies in the outpatient setting over recent years, but this has not consistently translated into inpatient use.

**Methods:**

An online survey was undertaken to understand the current use of technology to support inpatient diabetes care in the United Kingdom.

**Results:**

Responses were received from 42 different organizations representing 104 hospitals across the United Kingdom. Significant variation was found between organizations in the use of technology to support safe, effective inpatient diabetes care. Benefits of the use of technology were reported, and areas of good practice identified.

**Conclusion:**

Technology supports good inpatient diabetes care, but there is currently variation in its use. Guidance has been developed which should drive improvements in the use of technology and hence improvements in the safety and effectiveness of inpatient diabetes care. Key recommendations include implementation of this guidance (especially for continuous glucose monitoring), ensuring specialist support is available for the use of wearable diabetes technology in hospital, optimizing information sharing across the health care system, and making full use of data from networked glucose and ketone meters.

## Introduction

Day-to-day use of wearable diabetes technology such as insulin pumps and continuous glucose monitoring (CGM) has increased in the United Kingdom over recent years, partly related to recent guidelines which have led to improved access.^1,2^ Use of these technologies has been shown to improve safety for people with diabetes in community settings by reducing both HbA1c and hypoglycaemia.^3-5^ It is reasonable to think that the same features which lead to these improvements in the community could also lead to improved diabetes management in hospital. For those on interstitial glucose monitoring, these include ready access to glucose levels without the need for fingerpicking, and the ability for the person with diabetes (pwd) to set alerts to high and low readings. For those on insulin pumps, there is the benefit of greater flexibility in insulin delivery when compared with subcutaneous insulin injections. People with diabetes often report feeling unsafe in inpatient settings and most would feel more secure if allowed to continue to use the technology with which they are familiar to manage their diabetes.^6,7^ Ward staff, who are often unfamiliar with the devices used, can find it difficult to support this.

While wearable diabetes technologies carry clear potential for benefit, there are also other technologies that can help to improve the safety of inpatient diabetes care. These include electronic patient records (EPRs), electronic prescribing, and automated alerts for identifying people with diabetes on admission to hospital. However, experience shows that implementing such technologies can be challenging. While some data about technology use have been collected by national audits in England and Wales, we wished to discover in more detail the current state of the use of technology to support inpatient diabetes management across the United Kingdom. An in-depth survey was therefore designed to address this gap in current knowledge.

## Methods

The survey used the Jisc online survey platform (https://www.onlinesurveys.ac.uk) which has strict information security standards and is widely used by academic institutions. The survey aimed to look at all aspects of the journey of a pwd through their inpatient stay, and consider areas in which technology could be used to support inpatient diabetes care. The availability of specialist staff trained in the use of diabetes technology was also considered.

Questions were written and refined by representatives of Diabetes Technology Network UK (DTN-UK), part of the Association of British Clinical Diabetologists (https://abcd.care/dtn), and the Joint British Diabetes Societies for Inpatient Care (JBDS-IP) group, a UK group which develops guidance for diabetes management in inpatient settings (https://abcd.care/joint-british-diabetes-societies-jbds-inpatient-care-group). The survey link was distributed via electronic mailing lists for these groups, and all organizations providing inpatient care for people with diabetes were invited to contribute. The survey was open from 26 May 2022 to 30 June 2022, and repeated invitations were sent via email prompting potential participants to take part. Participation was voluntary, at the discretion of participating centers. The survey questions are available as an online appendix.

Results are reported as both proportions and percentages of respondents that gave a particular answer. Where not all respondents answered a particular question, the denominator used is the total number of responses to that question.

## Results

### Participation

Forty-eight responses were received from 42 different organizations across the four nations of the United Kingdom. These organizations represent a total of 104 UK hospitals, with the majority of organizations representing more than one hospital. Where more than one response was received from an organization, only the first response was reported. Twenty-three of 42 (54.8%) responses were completed by a consultant in diabetes, 15/42 (35.7%) by a diabetes specialist nurse, 3/42 (7.1%) by other doctors, and 1/42 (2.4%) by a specialist pharmacist. The location of responding organizations and the number of hospitals covered by the survey responses are summarized in Table 1. Responding organizations were of variable sizes, as can be seen both from the number of hospitals within each organization (Table 1) and the number of inpatient beds represented (Table 2). It should be noted that not all organizations responded to each question. Some questions had a lower response rate, making it more challenging to draw conclusions from the responses.

**Table 1.** Location and Number of Hospitals Within Each Responding Organization.

	Number of hospitals covered by survey responses						Total number of hospitals
	1	2	3	4	More than 4	Total	
England	8	10	5	4	0	27	59
Scotland	2	1	3	1	2	9	27
Northern Ireland	1	0	1	2	0	4	12
Wales	0	0	2	0	0	2	6
Total	11	11	11	7	2	42	104

**Table 2.** Number of Inpatient Beds in Responding Organizations.

	Number of inpatient beds			
	Less than 500	500-750	751-1000	More than 1000
Number of organizations	7	14	6	15

### Staffing and Staff Training

There was significant variation between organizations in the availability of specialist support for people with diabetes using wearable diabetes technology (summarized in Table 3). All responding organizations provided this support either always or sometimes on weekdays during normal working hours, but out-of-hours availability was much more variable. Where no support was available, a variety of different solutions to support people using diabetes technology in hospital were described. These included ensuring prior education to support inpatient self-management, the use of local and national guidelines, the use of insulin pump company helplines, and conversion to variable rate intravenous insulin infusion (VRIII) until specialist support is available.

**Table 3.** Availability of a Member of the Diabetes Specialist Team to Support People With Diabetes Using Wearable Technology in Hospital.

	Always	Sometimes	No
Weekdays Normal working hours	35/42 (83.3%)	7/42 (16.7%)	0/42 (0%)
Weekdays Outside normal working hours	7/42 (16.7%)	8/42 (19.0%)	27/42 (64.3%)
Weekends	6/41 (14.6%)	13/41 (31.7%)	22/41 (53.7%)

Support during normal working hours on weekdays was provided by diabetes specialist nurses in the vast majority (33/36, 91.7%) of organizations (Table 4). Diabetes specialist doctors were also involved in providing support during normal working hours on weekdays in just under half (17/36, 47.2%) of organizations. Others involved in providing support were diabetes specialist pharmacists, diabetes specialist dietitians, and diabetes support workers (band 4 staff with specific training in diabetes technology). Out-of-hours support was more commonly provided by diabetes specialist doctors, often in conjunction with general medical (nonspecialist) on-call work which may limit the time which can be allocated to specialist work.

**Table 4.** Which Team Members Are Available to Support People With Diabetes Using Wearable Technology in Hospital.

	Diabetes specialist nurse	Diabetes specialist doctor	Other
Weekdays Normal working hours	33/36 (91.7%)	17/36 (47.2%)	6/36 (16.7%)
Weekdays Outside normal working hours	4/14 (28.6%)	11/14 (78.6%)	1/14 (7.1%)
Weekends	11/18 (61.1%)	14/18 (31.7%)	1/18 (5.6%)

Diabetes specialist inpatient teams report having adequate training in the use of wearable diabetes technology (Table 5), although in a small number of centers, the team members on call out of hours may not yet be adequately trained.

**Table 5.** Familiarity With the Use of Wearable Diabetes Technologies for Staff Supporting People With Diabetes in Hospital.

	Expert	Familiar	Unfamiliar
Weekdays Normal working hours	21/39 (53.8%)	31/39 (79.5%)	0/39 (0%)
Weekdays Outside normal working hours	12/17 (70.6%)	9/17 (52.9%)	2/17 (11.8%)
Weekends	11/20 (55.0%)	15/20 (75.0%)	3/20 (15.0%)

### Wearable Diabetes Technology

Only 7/42 (16.7%) organizations have a specific policy in place for the use of CGM in people with diabetes admitted to hospital. Thirty-two of 35 (91.4%) of those without a specific policy report that they permit people with diabetes to use their own CGM devices when in hospital. The results of our survey support the suggestion that this is primarily to support self-management decisions. Thirty-four of 37 (91.9%) centers reported that fingerstick glucose monitoring would continue alongside CGM. Those organizations that do permit use of CGM often limit its use to specific scenarios. As can be seen in Table 6, there is significant variation in the use of CGM for these scenarios and overall permission for the use of CGM is low.

**Table 6.** Situations in Which Organizations Permit Continuous Glucose Monitoring Use in Hospital.

Labor, delivery, and the postpartum period	16/39 (41.0%)
Perioperative period—elective surgery	14/39 (35.9%)
During imaging investigations	7/39 (17.9%)
Other	5/39 (12.8%)
Perioperative period—emergency surgery	4/39 (10.3%)
Management of variable rate intravenous insulin infusions (VRIII)	3/39 (7.7%)
Critical care settings	1/39 (2.6%)

Thirty-five of 42 (83.3%) organizations reported that there are circumstances in which CGM would not be used. These circumstances are summarized in Table 7. Other situations in which CGM would not be used would be during labor, and surgeries above a defined duration (although this duration varies between centers). In these circumstances, all clinical decisions involving staff will be made using fingerprick monitoring, even when people with diabetes are permitted to use CGM in hospital to support self-management.

**Table 7.** Circumstances in Which Organizations Would Not Permit Continuous Glucose Monitoring Use in Hospital.

Magnetic resonance imaging scans	33/35 (94.3%)
Operations with diathermy	30/35 (85.7%)
Computed tomographic scans or other investigations using x-rays	21/35 (60.0%)
Other	5/35 (14.3%)

These limitations on the use of CGM are in place despite 26/42 (61.9%) respondents reporting benefits of using CGM over fingerprick glucose monitoring. Five of 42 (11.9%) reported no benefits and the remainder (11/42, 26.2%) did not know whether there was benefit or not. The benefits of using CGM in hospital are summarized in Table 8. Other reported benefits were for people with diabetes to be able to make timely decisions about self-management.

**Table 8.** Reported Benefits of Using Continuous Glucose Monitoring in Hospital.

Empowerment of people with diabetes	25/26 (96.2%)
More information to guide treatment decisions	19/26 (73.1%)
The ability to review results remotely	17/26 (65.4%)
Prevention of hypoglycaemia	17/26 (65.4%)
Reduced need for fingerstick monitoring	13/26 (50.0%)
Other	1/26 (3.8%)

Thirty-four of 41 (82.9%) respondents also reported that there had been particular challenges which had affected the use of CGM in hospital when compared with fingerstick monitoring. These are summarized in Table 9. Other barriers were a lack of national guidance on the use of CGM in the inpatient setting, discrepancies between CGM and capillary blood glucose readings, and the fact that CGM glucose results are not transferred into electronic record systems, bypassing safety systems supported by results from networked glucose and ketone monitors.

**Table 9.** Challenges Affecting the Use of Continuous Glucose Monitoring in Hospital Compared With Fingerstick Glucose Monitoring.

Staff unfamiliarity with devices	34/34 (100%)
Concerns about accuracy	22/34 (64.7%)
Clinical governance concerns including worries about indemnity	16/34 (47.1%)
Storage and prescription of devices	15/34 (44.1%)
Unclear indications for use	12/34 (35.3%)
Other	5/34 (14.7%)

In contrast with CGM, a greater proportion of organizations (27/42, 64.3%) reported having a policy for the use of personal insulin pump or hybrid closed loop (HCL) systems in people admitted to hospital. Where no specific policy was in place, 14/15 (93.3%) respondents reported that people would be permitted to use personal insulin pumps or HCL systems in hospital. Scenarios in which these technologies are used in hospital are summarized in Table 10. Personal insulin pump or HCL use was also permitted in other inpatient situations when the person was deemed capable to self-manage the pump.

**Table 10.** Situations in Which Personal Insulin Pumps and Hybrid Closed Loop Systems Are Used in Hospital Settings.

In the perioperative period—elective surgery	32/38 (84.2%)
During labor, delivery, and the postpartum period	31/38 (81.6%)
During imaging investigations	12/38 (31.6%)
In the perioperative period—emergency surgery	6/38 (15.8%)
Other	5/38 (13.2%)
In critical care settings	3/38 (7.9%)

Thirty-two of 40 (80.0%) respondents reported that there had been advantages to allowing people to use personal insulin pumps or HCL systems while in hospital. The main advantages reported were empowerment of people with diabetes and improvements in glycaemia. Twenty-six of 40 (65.0%) reported challenges to the use of these systems in hospital. The main challenges were with unfamiliarity of nonspecialist staff with the devices, and the potential for this putting them in conflict with people with diabetes.

### Diabetes Alerts

Seventeen of 42 (40.5%) organizations reported having a system to provide an automated flag to identify people with diabetes on admission. Twenty-four of 42 (57.1%) organizations did not have such a system. A number of sources were used to identify people with diabetes including links with local and national diabetes databases, primary care records, and retinal screening databases. Where automated identification of people with diabetes was not available, some organizations identified people with diabetes using compulsory questions in admission documentation, the prescription of glucose-lowering medications, and elevated blood glucose readings. A small proportion of organizations (12/42, 28.6%) have extended their systems to identify people at increased risk of experiencing diabetes-related harm when they are admitted to hospital.

Thirty of 41 (73.1%) organizations have an electronic system to refer people in hospital to the diabetes team. Nineteen of 29 (65.5%) report particular advantages to using an electronic referral system over previous systems. Nine of 29 (31.0%) organizations reported particular challenges with using an electronic referral system. The advantages and disadvantages reported are listed in Box 1.

**Box 1.** Reported Advantages and Disadvantages of Electronic Systems to Refer People With Diabetes in Hospital to the Diabetes Team.

Advantages	Disadvantages
• Streamlining of referral processes • Increased robustness of systems (no referrals being lost) • The ability to trigger quicker responses (both at the bedside and from the specialist team) • The ability to provide remote support • Improved prioritization • Ability to make earlier referrals (including out of hours) • Ability to audit referral activity including volume of referrals and diabetes inpatient team response time	• Information technology problems (eg, long login times, lack of ward computers) • Laborious setup processes • Problems with interoperability between systems • Inappropriate automated alerts (eg, neonatal hypoglycaemia to adult diabetes team)

Another means of referral to the diabetes team is to use the features of the web-linked glucose meters. This is discussed in the next section on inpatient monitoring.

### Inpatient Monitoring

Thirty-six of 42 (85.7%) responding organizations use blood glucose meters linked in an electronic network which allows results to be collected automatically by a central system. Where blood glucose meters are networked, the results are then made available using the organization’s usual system for viewing blood test results in 27/36 (75.0%) of cases. Diabetes teams have access to a database of results from networked meters in 29/36 (80.6%) of cases. These data are used for audit, quality improvement, or clinical care in 21/36 (58.3%) of organizations.

One significant potential advantage of networked blood glucose meters is their use in alerting staff to out-of-range results, both to support ward staff at the bedside and also to provide alerts remotely to the diabetes specialist team. This facility is not used widely in the organizations responding to our survey, and there is wide variation in the thresholds at which an alert is generated. Only 11/41 (26.8%) have alerts for clinical staff generated automatically for glucose readings below a certain threshold. Eight of nine (88.9%) respondents who gave further information report that alerts are generated for readings below 4.0 mmol/L, with 2 centers reporting these alerts are only generated for recurrent readings below this level. One of nine (11.1%) organization reported generating automated alerts for glucose readings below 3.0 mmol/L. Nine of 40 (22.5%) organizations generate automated alerts for glucose readings above a particular threshold, with the threshold varying from above 9.9 mmol/L to above 27.8 mmol/L.

All organizations use bedside capillary ketone meters, but these are only linked in a network in 32/42 (76.2%) of organizations. Ketone results are available to view in the organization’s usual system for viewing blood test results in 21/32 (65.6%) of cases. In only three of 40 (7.5%) organizations, there are automated alerts for ketone readings above a certain threshold. In two of three (75%) cases, this threshold is 1.5 mmol/L and in one in four (25%), the threshold is 3.0 mmol/L.

Thirty-three of 36 (91.7%) respondents using networked glucose or ketone meters reported advantages to using them. These advantages are summarized in Table 11. Other reported advantages were the ability to review historical data and the ability to assess strip usage and standardize quality control procedures.

**Table 11.** Advantages of Networked Glucose/Ketone Meters Compared With Previous Systems.

The ability to review results remotely	31/36 (86.1%)
The ability to analyze trends for individuals	28/36 (77.8%)
Improved accuracy of results recording	27/36 (75%)
The ability to access data for audit/clinical governance	27/36 (75%)
Earlier involvement of the diabetes team with people requiring input	24/36 (66.7%)
Other	2/36 (5.6%)

Thirty of 36 (83.3%) respondents also reported challenges with using networked meters. These are summarized in Table 12. Other issues reported were that the procedure involved to access the data is too complex to be used for patient care, there are problems with linking with EPR, there are issues with staff understanding the importance of entering correct details, and that inappropriate quality control for ketone testing is an increasing problem.

**Table 12.** Challenges With Networked Glucose/Ketone Meters Compared With Previous Systems.

Need to provide staff training	26/30 (86.7%)
Requirement for staff registration	24/30 (80.0%)
Untrained staff using emergency login details or those of other staff	24/30 (80.0%)
Problems with connectivity (including Wi-Fi)	16/30 (53.3%)
Increased complexity of the testing process	12/30 (40.0%)
Delays in ward staff acting on data	11/30 (36.7%)
Difficulties in connecting with the usual results viewer	11/30 (36.7%)
Other	4/30 (13.3%)

### Electronic Patient Records

There is also significant variation in the use of EPR systems. Thirty-nine of 42 responding organizations (92.9%) use some form of electronic system to record key information during an inpatient stay, but only 20/39 (51.3%) report having a single EPR system which combines information from multiple domains such as clinical notes, laboratory, and radiology results. Seven of 39 (17.9%) reported having multiple different electronic systems to record information including one recording clinical notes. Twelve of 39 (30.8%) reported multiple electronic systems for recording results but did not have an EPR for recording clinical notes.

Twenty of 42 (47.6%) responding organizations have a specific diabetes database, of which only 15/42 (35.7%) use their diabetes database for support of inpatients with diabetes. Where available, databases are used to identify people with diabetes on admission, to identify key features of a person’s diabetes history including usual medication and management strategies, and to identify previous results where these are not available via other means. They are also used to store information about diabetes management during an inpatient stay, in order for this to be available for future encounters with the diabetes team. On a national level, Scotland (SCI-diabetes) and Northern Ireland (NIEHCR) have a comprehensive nationwide electronic record system which also supports diabetes care in the inpatient setting.

### Other Benefits of Technology

Eleven of 42 (26.2%) responding organizations reported using technology in other ways to support inpatient diabetes care. Examples were the use of dashboards for quality improvement work, technology to allow remote communication with patients in ward rooms, an app to carry information about inpatient diabetes management, an alert for when young adults with diabetes are admitted, the use of temporary CGM in specific cases, the ability to review data remotely and support management across a wider geographical region, the ability to support diabetes management in a “hospital at home” setting^8^ and the use of machine learning to identify people at risk of hypoglycaemia.

## Discussion

This survey reports responses from organizations representing 104 UK hospitals. Participation was voluntary, which likely explains why participation was lower than that reported for the National Diabetes Inpatient Safety Audit (NDISA) for England and Wales as this is a mandatory audit.^9^ The results provide additional understanding regarding how technology is being used across the United Kingdom, as responses from Scotland and Northern Ireland are included. The questions asked in NDISA are fixed to allow for comparison from year to year, and this survey allowed the collection of additional data to further our understanding of this interesting topic.

The survey reveals significant variation in how technology is currently being used to support inpatient diabetes care in the United Kingdom. This variation exists for the provision of specialist support, the use of networked glucose and ketone meters, and the use of wearable diabetes technologies, as well as for domains such as the use of electronic referral systems, EPRs, and diabetes databases. Given the reported benefits of using technology to support inpatient diabetes management, this variation in the use of technology is likely to contribute to variations in the safety and effectiveness of inpatient care. It is imperative that we work to minimize this variation. The results have been used to identify best practice, which has been developed into national guidance^10-12^ to be shared widely. Such guidance should be used to promote the widespread use of technology with the intention of improving inpatient safety for people with diabetes.

The survey demonstrates that the primary source of support for people in hospital using wearable diabetes technology is diabetes specialist nursing staff. The NDISA for England and Wales reveals that only 32% of organizations have weekend diabetes nurse specialist cover,^9^ and so it was not surprising to find that there is a lack of experienced staff to support those using wearable technologies at weekends. The deficiency was even greater outside normal working hours when diabetes specialist nursing staff are usually not available and support is mainly provided by diabetes medical staff, often when they have other on-call commitments. Most people using wearable technologies effectively use these devices to self-manage independently, but it is important to recognize that this is not always the case. Furthermore, even those who usually manage independently may find this more difficult during illness. As such, all people using wearable technology should ideally be assessed by the diabetes team to ensure that it is safe for them to continue using the technology, and that they have the support they need to do so. Some organizations may struggle to provide this support out of hours. One solution, which could address expert staff deficiencies outside normal working hours, would be to consider a regional or national helpline staffed by appropriately trained health care professionals. This survey found that support can be provided by people with adequate training from a variety of different health care backgrounds, not just medicine and nursing, and this could also be the case for such an out-of-hours service.

While a limited number (16.7%) of organizations have a specific policy in place for the use of CGM in hospital, the vast majority (91.4%) of those who do not will permit people with diabetes to use their own CGM. The results of our survey suggest that CGM is being used to support self-management, rather to support diabetes management advice from staff. This is despite the fact that organizations report a beneficial effect of CGM in providing more information to guide treatment decisions and the prevention of hypoglycaemia as well as the empowerment of people with diabetes. It is important to note that current safety measures often use data from networked glucose meters. In order for these benefits to continue, fingerstick glucose monitoring is usually continued alongside CGM use, until methods can be found to use CGM data in safety systems. One barrier to CGM use in inpatient settings is the lack of a current national guidance, and this will be addressed by guidance currently being developed by JBDS-IP and DTN-UK.^10^

National guidance, developed by DTN-UK, already exists for the use of personal insulin pumps in hospital,^13^ which may explain why a much higher proportion (64.3%) of respondents to our survey reported having guidance in place when compared with CGM. Almost all centers would permit the use of personal insulin pumps or HCL systems in an inpatient setting, and the development of updated guidance including HCL systems^11^ should further support their use in hospital. The main advantages of insulin pumps and HCL systems were seen as empowerment of people with diabetes and improvements in glycemia, with the main barrier being the unfamiliarity of nonspecialist staff with the devices. This could put staff in conflict with people with diabetes and contribute to a pwd not feeling safe in an inpatient setting. Access to specialist support round the clock will help to mitigate this risk. Education of nonspecialist staff may also be helpful to increase familiarity.

National databases in Scotland and Northern Ireland allow information from the community to be accessed relatively easily in hospital, permitting a continuity of information which can support safe care across all aspects of a person’s care. In England and Wales, less than half of organizations have a diabetes-specific database, and where these exist they are only used to support inpatient care in 65.2% of cases. Most responding organizations (92.9%) use electronic systems to record key information during an inpatient stay, but only just more than half have a single electronic record which combines information from multiple domains. The recent NDISA audit found that only 12% of organizations had all of the following:^9^ EPRs, electronic prescribing records, and Web-linked glucose meters.

Working to optimize information sharing is key to making these data accessible for inpatient care and therefore making best use of the information that is collected and stored to support safe and effective care.

The Getting it Right First Time (GIRFT) program in England recommends having a system to provide an automated flag to identify people with diabetes on admission to hospital.^14^ Only 40.5% of organizations reported having such a system, compared with 51% in England and Wales in the NDISA report published in July 2022.^9^ Similarly, 28.6% in our survey have a system to identify people at risk of diabetes-related harm on admission to hospital, compared with 56% in the NDISA report. Organizations should continue to aim to increase the recognition of people with diabetes on admission, and, in particular, those at increased risk of harm, as this will allow measures to be put in place to mitigate this risk and improve the safety and effectiveness of inpatient diabetes care. The reasons for the discrepancies between our survey and the NDISA report are not clear. It may be simply that different organizations responded to the two surveys or different members of the diabetes team received and responded to the surveys. The current survey covered a wider geographical area, although expanding the pool to include Scotland and Northern Ireland might not be expected to have a negative effect on these particular parameters given the national diabetes databases they have available.

In all, 85.7% of organizations use glucose meters linked in a network, which is a similar figure to that reported in the GIRFT diabetes report.^14^ As noted in that report, networked glucose meters have been shown to reduce severe hypoglycemia by more than 45% as part of a wider suite of measures. In the GIRFT report, of the 71% of trusts with networked meters, 65% reported being able to use alerts with networked meters. This corresponds to 46% of all trusts having systems to identify out-of-range glucose readings. In our survey, 91.7% of those using networked glucose or ketone meters reported benefits from using them. However, in contrast to the GIRFT findings, only 26.8% of organizations reported using the networked meters to generate alerts for out-of-range glucose readings. Furthermore, only 58.3% of organizations that had access to the database of results from networked meters were using these for audit or clinical governance purposes. The results suggest that centers are not currently maximizing the opportunities offered by currently available technologies to support safe inpatient care.

All organizations reported using capillary ketone meters. This is likely related to the fact the JBDS guidance for the management of diabetic ketoacidosis^15^ has recommended the use of capillary ketones to guide treatment since 2012. This guidance differs from that used in other countries, including the United States, where capillary ketone measurement is less common. Only three of 40 (7.5%) of organizations use these meters to generate alerts for out-of-range values, meaning that there is also significant scope to improve the use of ketone meters in supporting safe inpatient diabetes management.

**Box 2.** Key Recommendations based on the survey results.

• Implement national guidance for the use of technology in hospitals, including specific guidance for the use of continuous glucose monitoring in hospital settings • Ensure that support is available for people with diabetes to make use of wearable technology in hospital ○ Consider regional or national helplines to support hospitals that do not have specialist support available in person outside normal working hours • Optimize information sharing between general practice and hospital record systems ○ Use collected information to identify people with diabetes on admission to hospital, including specific flags for those at high risk • Optimize use of data from networked glucose and ketone meters ○ Ensure data are used for audit and quality improvement

The strength of our survey is that it captures detailed information from 104 hospitals of varying sizes across the United Kingdom. Respondents were asked about a wide variety of ways in which technology could benefit inpatient diabetes care, and an attempt was made to understand both the advantages and disadvantages of the technologies involved. It provides baseline data against which both future responses and international responses could be compared.

The main limitation is that the survey does not represent all centers in the United Kingdom, and so (as with all surveys) the results are limited to those willing to respond. While it may be expected that respondents would be more likely to be using technology successfully, this was not the case when we were able to compare results from our survey with results from a recent national audit report. Accepting that our survey may not represent the full state of technology use to support inpatient diabetes care in the United Kingdom, it offers significant insight into current provision and also suggestions as to how the situation could be improved.

## Conclusion

The results of the survey show significant variation in the use of technology to support safe inpatient diabetes care in the United Kingdom. It is positive to see that there are already pockets of good practice, but considerably more can be done to make best use of existing technology. Barriers to the uptake of technology are often shared. Sharing of best practice, including how to overcome these common barriers, should improve the inpatient experience for people with diabetes across the board. The development of national guidance^9-11^ is essential to drive ongoing improvements in technology use, and through these improvements in inpatient diabetes care.

## Supplemental Material

sj-pdf-1-dst-10.1177_19322968231161076 – Supplemental material for Variation in the Current Use of Technology to Support Diabetes Management in UK Hospitals: Results of a Survey of Health Care Professionals Click here for additional data file. Supplemental material, sj-pdf-1-dst-10.1177_19322968231161076 for Variation in the Current Use of Technology to Support Diabetes Management in UK Hospitals: Results of a Survey of Health Care Professionals by Alistair Lumb, Shivani Misra, Gerry Rayman, Parizad Avari, Daniel Flanagan, Pratik Choudhary and Ketan Dhatariya in Journal of Diabetes Science and Technology
