# Supplementary material for: Variation in the Current Use of Technology to Support Diabetes Management in UK Hospitals: Results of a Survey of Health Care Professionals
Source: J Diabetes Sci Technol. 2023 Mar 22;17(3):733–41. doi: 10.1177/19322968231161076 (PMC10210106; doi:10.1177/19322968231161076)
Supplement: sj-pdf-1-dst-10.1177_19322968231161076 – Supplemental material for Variation in the Current Use of Technology to Support Diabetes Management in UK Hospitals: Results of a Survey of Health Care Professionals [file sj-pdf-1-dst-10.1177_19322968231161076.pdf]

JBDS DTN inpatient technology survey

Page 1: Introduction

Why are we doing this survey?

Providing safe and effective diabetes care for people admitted to hospital requires a clinical team with specialist knowledge and skills. We are interested in how technology is being used to support the work of these teams.

Some technologies, such as electronic prescribing systems and electronic patient records, are well established. Others, such as continuous glucose monitors, are less well established in hospitals but offer significant oppportunities to improve safety.

The aim of this survey is to establish current practice. How are diabetes teams making use of available technologies to improve diabetes care? What have been the advantages and what are the challenges?

We would like to hear from as many centres as possible in the UK who are providing inpatient diabetes care. Only one response is required per centre. Please consider completing as a team. The survey should take around 20 minutes to complete in total.

Page 2: About you

1. Your role ✱ *Required*

Please select no more than 1 answer(s).

- ☐ Diabetes Specialist Nurse
- ☐ Diabetes Specialist Dietitian
- ☐ Podiatrist
- ☐ Consultant in Diabetes
- ☐ Specialist trainee in Diabetes/Endocrinology
- ☐ Other doctor
- ☐ Other

1.a. If you selected Other, please specify:

2. Your location ✱ *Required*

- ◯ England
- ◯ Northern Ireland
- ◯ Scotland
- ◯ Wales
- ◯ Other

2.a. If you selected Other, please specify:

3. What is the name of the Organisation (eg Trust, Health Board or other provider) where you work?

4. What is the name of the hospital where you work (if different)?

5. Are the policies and guidelines used in your hospital used in other hospitals within your organisation (eg across a Trust with multiple hospital sites)?

- Yes
- No
- Don't know

5.a. If yes, how many hospital sites are covered by your answers?

- 1
- 2
- 3
- 4
- More than 4

6. How many inpatient beds are covered by your answers?

- Under 500

- ☐ 500-750
- ☐ 751-1000
- ☐ Over 1000

Page 3: Inpatient diabetes care - staffing

7. Is there at least one member of the diabetes specialist team available to support people using diabetes technology in hospital during normal working hours on weekdays (Monday to Friday)?

- Always
- Sometimes
- No

7.a. If always or sometimes, please briefly describe all people who may fill this role (For example "A member of the inpatient nursing team with specific training")

7.b. If always or sometimes, what is their level of training in the use of wearable diabetes technologies such as insulin pumps and continuous glucose monitors (select all that may apply for members of staff covering this role)?

- Expert
- Familiar
- Unfamiliar

7.c. If no, how would people with diabetes using diabetes technology be supported?

8. Is there at least one member of the diabetes specialist team available to support

people using diabetes technology in hospital outside normal working hours (Monday to Friday)?

- ◯ Always
- ◯ Sometimes
- ◯ No

8.a. If always or sometimes, please briefly describe all people who may fill this role (For example "A member of the inpatient nursing team with specific training")

8.b. If always or sometimes, what is their level of training in the use of wearable diabetes technologies such as insulin pumps and continuous glucose monitors (select all that may apply for members of staff covering this role)?

- ☐ Expert
- ☐ Familiar
- ☐ Unfamiliar

8.c. If no, how would people with diabetes using diabetes technology be supported?

9. Is there at least one member of the diabetes specialist team available to support people with diabetes using diabetes technology in hospital at weekends (Saturday/Sunday)?

- ○ Always
- ○ Sometimes
- ○ No

9.a. If always or sometimes, please briefly describe all people who may fill this role (For example "A member of the inpatient nursing team with specific training")

9.b. If always or sometimes, how would you describe the level of training of these inpatient staff with diabetes technology such as insulin pumps and continuous glucose monitors (select all that may apply)?

- ☐ Expert
- ☐ Familiar
- ☐ Unfamiliar

9.c. If no, how would people with diabetes using diabetes technology be supported at weekends?

Page 4: Electronic patient record

For the purpose of this survey, an electronic patient record (EPR, alternatively electronic medical record, EMR, or electronic health record, EHR) is an electronic record which contains information about an individual's health history such as such as diagnoses, medicines, tests, allergies, immunizations, and treatment plans, and which replaces paper medical notes.

10. Does your hospital use electronic systems to record key information during an inpatient stay?

- A single electronic patient record system which combines information from multiple domains (eg clinical notes, laboratory results, radiology results)
- Multiple separate electronic systems (eg laboratory results, radiology results) including one recording clinical notes
- Multiple separate electronic systems (ag laboratory results, radiology results) not including one recording clinical notes
- No
- Don't know

11. If you use an electronic system for recording clinical notes, please provide as much information as you can about the name of the system and the supplier (for commercial systems) or whether it was developed in house

12. If you use an electronic system for recording clinical notes, is this used exclusively for patient records, or in combination with paper records?

- ○ Electronic system alone
- ○ Combination of electronic system and paper records
- ○ Don't know

Page 5: Admission to hospital

13. Does your hospital have a system which provides an automated flag to identify people with diabetes on admission to hospital?

- Yes
- No
- Don't know

13.a. If yes, how are people with diabetes identified?

- Retinal screening database
- Primary care record
- Local diabetes database
- Don't know
- Other

13.a.i. If you selected Other, please specify:

14. Does your hospital have a system to identify people on admission who are at particularly high risk of diabetes related harm (eg with characteristics assocated with increased risk of DKA or inpatient hypoglycaemia)?

- Yes
- No
- Don't know

15. Does you hospital have a system allowing access to diabetes clinic letters when a person with diabetes is admitted to hospital?

- ○ Yes
- ○ No
- ○ Don't know

15.a. If yes, who can access them (tick all that apply)?

- ☐ Specialist diabetes medical team
- ☐ Ward medical team
- ☐ Specialist diabetes nursing team
- ☐ Ward nursing team
- ☐ Don't know

Page 6: Links with primary care records

16. Are you able to view primary care records from within your organisation, even when primary care providers (eg GP surgeries) are closed?

- ○ Able to view fully
- ○ Able to view partially
- ○ No
- ○ Don't know

16.a. If yes, how is this done?

- ☐ Accessed via a linked hospital system
- ☐ Separate access to primary care record system (eg SystmOne, EMIS)
- ☐ Don't know
- ☐ Other

16.a.i. If you selected Other, please specify:

____

16.b. Can you send information electronically about the care of people with diabetes directly into primary care record systems?

- ○ Yes
- ○ No
- ○ Don't know

16.b.i. If yes, how is this done?

____

Page 7: Electronic Prescribing and Medicines Administration (ePMA)

17. Does your organisation use an electronic Prescribing and Medicines Administration (ePMA) system?

- ○ Yes
- ○ No
- ○ Don't know

17.a. If yes, which system is used?

____

18. Can you use this system to prescribe the following (select all that apply)?

- ☐ Oral glucose lowering agents
- ☐ Injectable non-insulin glucose lowering agents (eg GLP-1RA)
- ☐ Subcutaneous insulin
- ☐ Intravenous insulin
- ☐ Intravenous fluid
- ☐ Intramuscular glucagon

19. Thinking specifically about prescribing medications for people with diabetes, have there been specific advantages to using ePMA over previous systems?

- ○ Yes
- ○ No
- ○ Don't know

19.a. If yes, what have the advantages been (select all that apply)?

- [ ] Automated safety alerts for prescribers
- [ ] Ability to prescribe remotely
- [ ] Ability to view prescriptions remotely
- [ ] Ability to search prescriptions electronically
- [ ] Other

19.a.i. If you selected Other, please specify:

20. Thinking specifically about prescribing medications for people with diabetes, have there been specific challenges to using ePMA compared with previous systems?

- ○ Yes
- ○ No
- ○ Don't know

20.a. If yes, what have the challenges been?

- [ ] Prescribing iv insulin in DKA/HHS/VRIII
- [ ] Prescribing iv fluid in DKA/HHS/VRIII
- [ ] Confusion between insulins where names look/sound similar
- [ ] Selection of correct device for insulin delivery
- [ ] Other

20.a.i. If you selected Other, please specify:

Page 8: Diabetes database

For the purposes of this questionnaire, a diabetes database refers to a system which can identify people with diabetes seen by your service (inpatient or outpatient) and which can record relevant clinical parameters to support both clinical care and audit.

21. Does your organisation have a specific diabetes database?

- Yes
- No
- Don't know

21.a. If yes, is this a commercial product or developed locally?

- Commercial product
- Developed locally
- Don't know

21.a.i. If a commercial product then please provide as much information as you can about the name of the product and its supplier

22. If you have a diabetes database, is this used to support **inpatient** diabetes care?

- Yes
- No
- Don't know
- Not applicable

22.a. If yes, how this is done?

Page 9: Point of care monitoring

23. Does your organisation use capillary blood glucose meters which are linked in a network?

- Yes
- No
- Don't know

23.a. If networked meters are used, are you able to view the results using your usual system for viewing blood test results?

- Yes
- No
- Don't know

23.b. If networked meters are used, is the data transferred into your Laboratory Information Management System (LIMS - this will be a system used by the Biochemistry Laboratory)?

- Yes
- No
- Don't know

23.c. If networked meters are used, the data is usually transferred to some form of database (this may include an electronic patient record). Do you have access to this database?

- Yes
- No
- Don't know

23.c.i. If you have access to the database, do you use the data for audit/quality improvement or clinical care?

- Yes
- No
- Don't know

24. Does your organisation use capillary blood ketone meters?

- Yes
- No
- Don't know

24.a. If yes, are these meters linked in a network?

- Yes
- No
- Don't know

24.b. If networked meters are used, are you able to view the results using your usual system for viewing blood test results?

- Yes
- No
- Don't know

24.c. If networked meters are used, is the data transferred into your Laboratory Information Management System (LIMS)?

- Yes
- No

- Don't know

25. Have there been any particular advantages to using networked blood glucose/ketone meters over previous systems?

- Yes
- No
- Don't know

25.a. If yes, what have the advantages been? (select all that apply)

- [ ] Earlier involvement of the diabetes team with people requiring input
- [ ] The ability to review results remotely
- [ ] An improvement in accuracy of results recording (right patient, right result, right time)
- [ ] The ability to analyse trends for individuals
- [ ] The ability to access data for audit/clinical governance purposes
- [ ] Other

25.a.i. If you selected Other, please specify:

26. Have there been any particular challenges with using networked blood glucose/ketone meters compared with previous systems?

- Yes

- No
- Don't know

26.a. If yes, what have the challenges been? (Select all that apply)

- Increased complexity of the testing process
- Requirement for staff registration
- Need to provide staff training
- Untrained staff using emergency login details (eg barcodes) or those of other staff
- Delays in ward staff acting on data
- Difficulties in connecting with the usual results viewer
- Problems with connectivity (including wifi)
- Other

26.a.i. If you selected Other, please specify:

27. Which company makes the capillary blood glucose meters that you use?

28. Which company makes the capillary blood ketone meters that you use (if different)?

29. Does your organisation use a point of care HbA1c machine?

- Yes for both inpatients and outpatients
- Yes for inpatients only
- Yes for outpatients only
- No
- Don't know

Page 10: Electronic referral systems and automated alerts

30. Does your organisation have an electronic system to refer people in hospital to the diabetes team?

- ○ Yes
- ○ No
- ○ Don't know

31. Are alerts generated automatically from blood glucose readings below a specified threshold?

- ○ Yes
- ○ No
- ○ Don't know
- ○ Not applicable

31.a. If yes, what is this threshold?

[ ]

31.b. If yes, who receives these alerts (select all that apply)?

- ☐ Local ward staff
- ☐ Diabetes specialist team
- ☐ Other

31.b.i. If you selected Other, please specify:

[ ]

32. Are alerts generated automatically from blood glucose readings above a specified threshold?

- ○ Yes
- ○ No
- ○ Don't know
- ○ Not applicable

32.a. If yes, what is this threshold?

32.b. If yes, who receives these alerts (select all that apply)?

- ☐ Local ward staff
- ☐ Diabetes specialist team
- ☐ Other

32.b.i. If you selected Other, please specify:

33. Are referrals generated automatically from blood ketone levels above a specified threshold?

- ○ Yes
- ○ No
- ○ Don't know
- ○ Not applicable

33.a. If yes, what is this threshold?

33.b. If yes, who receives these alerts (select all that apply)?

- ☐ Local ward staff
- ☐ Diabetes specialist team
- ☐ Other

33.b.i. If you selected Other, please specify:

34. Have there been any particular advantages to using an electronic referral system over previous systems?

- ○ Yes
- ○ No
- ○ Don't know
- ○ Not applicable

34.a. If yes, what have the advantages been?

35. Have there been any particular challenges to using an electronic referral system

compared with previous systems?

- Yes
- No
- Don't know
- Not applicable

35.a. If yes, what have the challenges been?

36. Has using an electronic referral system allowed monitoring of response times for referrals?

- Yes
- No
- Don't know
- Not applicable

Page 11: Continuous glucose monitors in hospital

37. Does your organisation have a policy for the use of continuous glucose monitors (eg Freestyle Libre, Dexcom G6, Glucomen Day) in people with diabetes admitted to hospital?

- Yes
- No
- Don't know

38. Where no specific policy is available, are people with diabetes who are able to self-manage their diabetes permitted to continue to use their own continuous glucose monitoring devices while in hospital?

- Yes
- No
- Don't know
- Not applicable

38.a. If yes, would fingerprick monitoring continue alongside the use of a continuous glucose monitor?

- Yes
- No
- Don't know

39. Does your organisation allow the use of continuous glucose monitors in specific scenarios? Please select all that apply

- ☐ Management of Variable Rate Intravenous Insulin Infusions

- ☐ In the peri-operative period - elective surgery
- ☐ In the peri-operative period - emergency surgery
- ☐ In critical care settings
- ☐ During labour, delivery and the postpartum period
- ☐ During imaging investigations
- ☐ Other
- ☐ None of the above

39.a. If you selected Other, please specify:

40. Are there circumstances in which a continuous glucose monitoring device would not be used?

- ○ Yes
- ○ No
- ○ Don't know

40.a. If yes, then what circumstances (select all that apply)?

- ☐ Operations with diathermy
- ☐ CT scans or other investigations using X-rays
- ☐ MRI scans
- ☐ Other

40.a.i. If you selected Other, please specify:

41. Have there been any particular advantages of using continuous glucose monitors in hospital over fingerprick glucose monitoring?

- ◯ Yes
- ◯ No
- ◯ Don't know

41.a. If yes, what have the advantages been (select all that apply)?

- ☐ Reduced need for finger stick monitoring
- ☐ More information to guide decisions about treatment
- ☐ The ability to review results remotely
- ☐ Prevention of hypoglycaemia
- ☐ Empowerment of people with diabetes
- ☐ Other

41.a.i. If you selected Other, please specify:

42. Have there been any particular challenges which have affected the use of continuous glucose monitors in hospital in place of fingerprick glucose monitoring?

- ◯ Yes
- ◯ No
- ◯ Don't know

42.a. If yes, what have the challenges been (select all that apply)?

- ☐ Concerns about accuracy
- ☐ Staff unfamiliarity with devices
- ☐ Unclear indications for use
- ☐ Storage and prescription of devices
- ☐ Clinical governance concerns such as worries about indemnity
- ☐ Other

42.a.i. If you selected Other, please specify:

Page 12: Insulin pumps in hospital

43. Does your organisation have a policy for the use of personal insulin pumps or hybrid closed-loop systems in people with diabetes admitted to hospital?

- Yes
- No
- Don't know

44. Where no specific policy is available, are people who are able to self-manage their diabetes permitted to continue to use their own personal insulin pump or hybrid closed-loop system while in hospital?

- Yes
- No
- Don't know
- Not applicable

45. Does your organisation allow the use of personal insulin pumps or hybrid closed-loop systems in specific scenarios? Please check all that apply

- [ ] In the peri-operative period - elective surgery
- [ ] In the peri-operative period - emergency surgery
- [ ] During labour, delivery and the postpartum period
- [ ] In critical care settings
- [ ] During imaging investigations
- [ ] Other

45.a. If you selected Other, please specify:

46. Have there been any particular advantages in allowing people with diabetes to continue to use personal insulin pumps or hybrid closed-loop systems while in hospital?

- Yes
- No
- Don't know

46.a. If yes, what have the advantages been?

47. Have there been any particular challenges in allowing people with diabetes to continue to use personal insulin pumps or hybrid closed-loop systems while in hospital?

- Yes
- No
- Don't know

47.a. If yes, what have the challenges been?

Page 13: Other information

48. Does your organisation use technology in any other way to support inpatient diabetes care?

- Yes
- No

48.a. If yes, please describe how else technolgy is used to support inpatient diabetes care

Page 14: Sharing best practice

49. Where you have developed local guidelines, or developed local adaptations to commercially available systems, would you be willing to be contacted about the possibility of sharing these developments with other centres?

- Yes
- No

49.a. If yes, please leave your email address below. This will **only** be used to contact you for this purpose. If you would prefer not to leave your email address here, but are still happy to share, please contact the survey team directly on alistair.lumb@ouh.nhs.uk

Page 15: Final page

Many thanks for taking the time to complete this survey. This will be the first insight into how technology is being used to drive improvements in inpatient diabetes care in the UK, and will be used to support the development of guidance and sharing of current best practice.
